# Expression of Cell Competition Markers at the Interface between p53 Signature and Normal Epithelium in the Human Fallopian Tube

**DOI:** 10.1371/journal.pone.0156069

**Published:** 2016-06-03

**Authors:** Masahiko Kito, Daichi Maeda, Yukitsugu Kudo-Asabe, Naoki Sato, Ie-Ming Shih, Tian-Li Wang, Masamitsu Tanaka, Yukihiro Terada, Akiteru Goto

**Affiliations:** 1 Department of Cellular and Organ Pathology, Graduate School of Medicine, Akita University, Akita, Japan; 2 Department of Obstetrics and Gynecology, Graduate School of Medicine, Akita University, Akita, Japan; 3 Department of Pathology, Oncology and Gynecology and Obstetrics, Johns Hopkins Medical Institutions, Baltimore, MD, United States of America; 4 Department of Molecular Medicine and Biochemistry, Graduate School of Medicine, Akita University, Akita, Japan; Hokkaido University, JAPAN

## Abstract

There is a growing body of evidence regarding cell competition between normal and mutant mammalian cells, which suggest that it may play a defensive role in the early phase of carcinogenesis. *In vitro* study in the past has shown that overexpression of vimentin in normal epithelial cells at the contact surface with transformed cells is essential for the cell competition involved in epithelial defense against cancer. In this study, we attempted to examine cell competition in human tissue *in vivo* by investigating surgically resected human fallopian tubes that contain p53 signatures and serous tubal intraepithelial lesions (STILs), a linear expansion of p53-immunopositive/*TP53* mutant tubal epithelial cells that are considered as precursors of pelvic high grade serous carcinoma. Immunofluorescence double staining for p53 and the cell competition marker vimentin was performed in 21 sections of human fallopian tube tissue containing 17 p53 signatures and 4 STILs. The intensities of vimentin expression at the interface between p53-positive cells at the end of the p53 signature/STIL and adjacent p53-negative normal tubal epithelial cells were compared with the background tubal epithelium. As a result, the average vimentin intensity at the interfaces relative to the background intensity was 1.076 (95% CI, 0.9412 – 1.211 for p53 signature and 0.9790 (95% CI, 0.7206 – 1.237) for STIL. Thus, it can be concluded that overexpression of the cell competition marker vimentin are not observed in human tissue with *TP53* alterations.

## Introduction

Normal and transformed epithelial cells often compete with each other for cell survival, a phenomenon called cell competition.[1,2] Cell competition was initially confirmed in *Drosophila* in 1975 and has been recently shown in mammalian cells.[3–7] Cell competition is now considered a unique type of short-range cell-to-cell communication that may take place during the early stages of carcinogenesis.[7,8] Genes such as *myc*, *scribble*, *sparc* and *mahjong* have been shown to play an important role in this process.[9–13] Based on the results of *in vitro* studies of mammalian epithelial cell competition, which demonstrated that “loser cells” are extruded or undergo apoptosis, while “winner cells” proliferate and fill the vacant spaces, the novel concept of “epithelial defense against cancer (EDAC)” has been introduced.[8] In fact, Kajita et al. extensively analyzed the molecular mechanism of cell competition between normal and RASV12-transformed or Src-transformed epithelial cells and showed that overexpression of vimentin (VMN) and filamin-A (FLNA) in normal epithelial cells at the contact surface with transformed cells are essential factors for the cell competition involved in EDAC.[8] Although these observations provided new insights in the field of cancer research, cell competition between normal and mutant cells has not been assessed or validated in human tissue *in vivo*.

**Fig 1:**
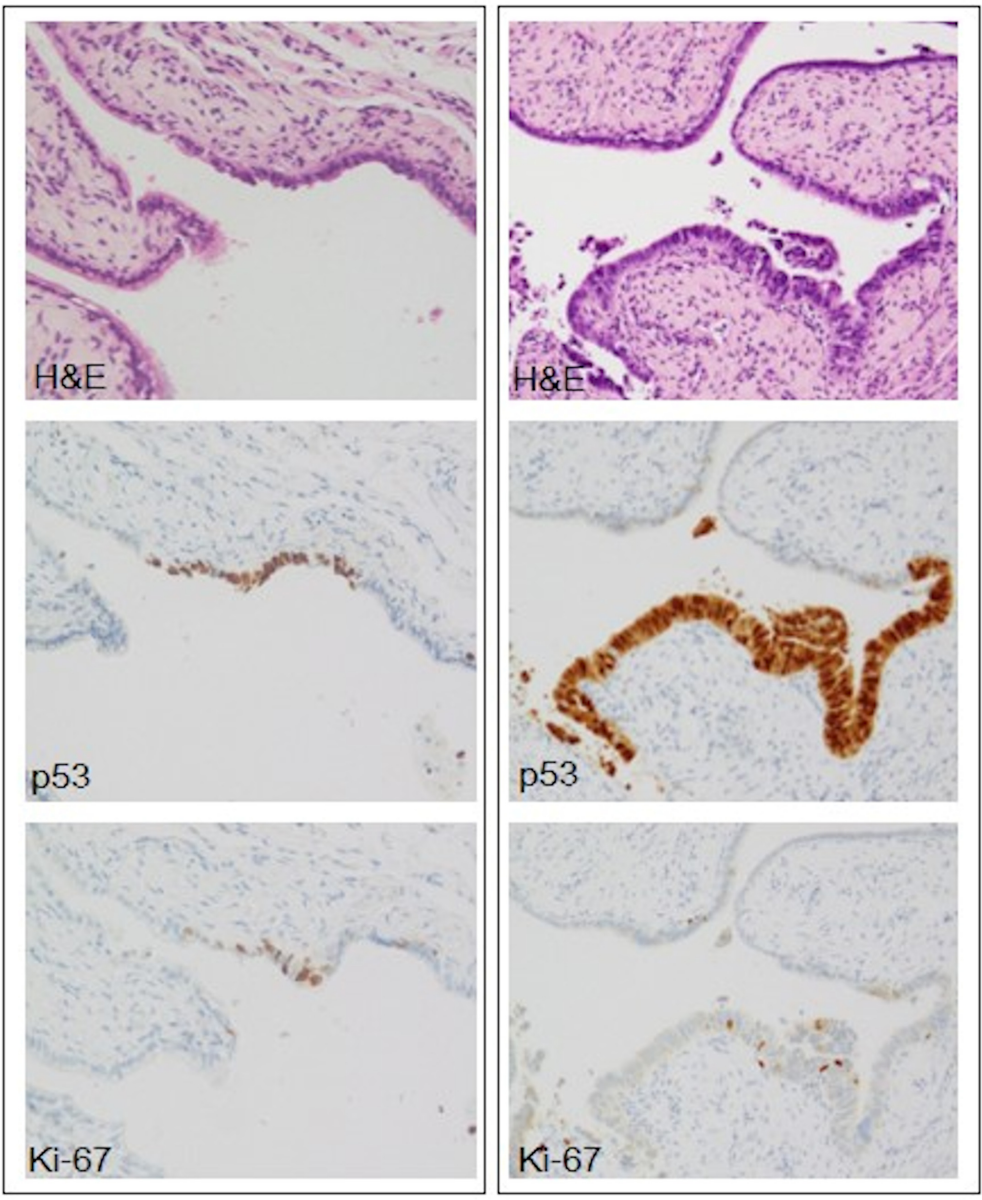
Histological features of serous tubal intraepithelial lesion (STIL). Two representative STILs (right and left) are shown. STIL on the right is a linear expansion of p53-positive tubal epithelial cells that show no morphological atypia, but increased Ki-67 labelling index (≥10%). STIL on the left consists of p53-positive tubal cells with mild to moderate morphological atypia. It is suspicious for serous tubal intraepithelial carcinoma on morphological basis. However, its Ki-67 labelling index is low (< 10%).

To examine the expression of cell competition markers at the interface between normal and transformed epithelial cells, we focused on studying the p53 signature of the human fallopian tube, a putative precursor of pelvic high-grade serous carcinoma.[14,15] The p53 signature is defined as a linear expansion of more than 12 p53-immunopositive/*TP53* mutant benign-appearing tubal secretory epithelial cells which show no increase in proliferative activity. It can occasionally be found even in non-cancer patients.[14,16] We also examined human serous tubal intraepithelial lesion (STIL),another tubal lesion with *TP53* altertation which is more advanced than p53 signature in tumor progression. STIL is a heterogeneous entity which encompasses morphologically bland p53-positive lesion that show increased proliferative activity, and lesion suspicious for serous tubal intraepithelial carcinoma on morphology, but shows low proliferative activity (Fig 1). We considered the p53 signature, along with STIL, to be the most suitable for our study, because it is immunohistochemically detectable, and its role in early carcinogenesis has been well documented.

## Materials and Methods

### Case Selection

**Fig 2:**
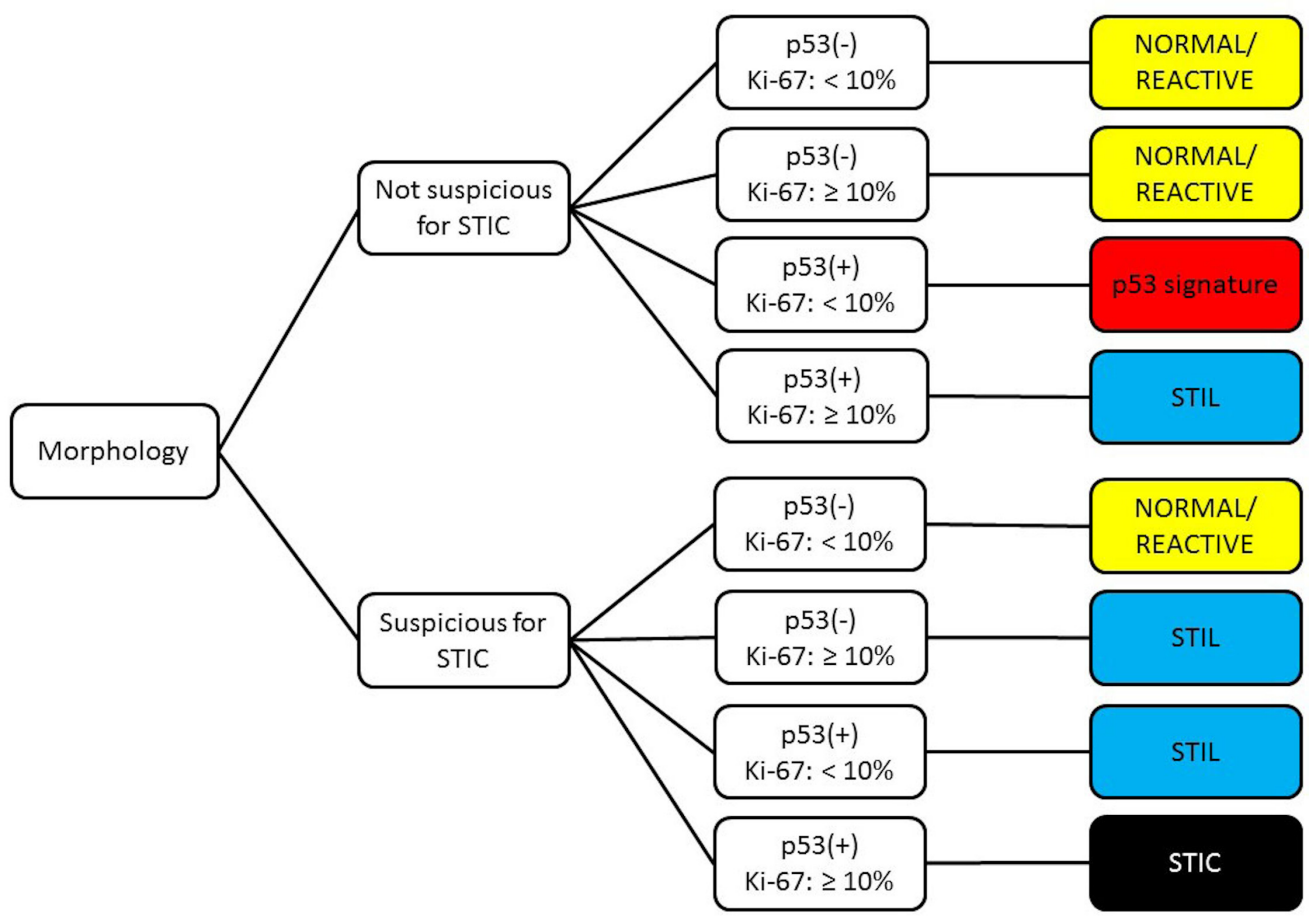
The diagnostic algorithm of tubal epithelial lesions with p53 alterations.[16,17]. STIC; serous tubal intraepithelial carcinoma, STIL; serous tubal intraepithelial lesion.

Between 2014 and 2015, we performed *in toto* sectioning of fallopian tubes according to the sectioning and extensively examining the fimbriated end (SEE-FIM) protocol in approximately 100 surgically resected gynaecological cases, and one of the authors (D.M.) routinely assessed the presence of p53 abnormalities in these specimens. The diagnoses of p53 signature and STIL were made based on the diagnostic algorithm described previously (Fig 2).[11,12] For this study, we selected 21 formalin-fixed paraffin-embedded sections of fallopian tube tissue from 18 patients, containing 17 p53 signatures and 4 STILs total. Tubal lesions that were morphologically definite for serous tubal intraepithelial carcinoma were not included. The demographics of the patients enrolled in this study are summarized in Table 1.

**Table 1:** Clinicopathological features of cases evaluated in this study.

Case	Age	Lesions evaluated	Location (P; proximal fallopian tube, D; distal fallopian tube, F; fimbriated end)	Pelvic disease the surgery was performed for
	32	p53 signature	D	Hydrosalpinx
	41	p53 signature	F	Hydrosalpinx
	44	p53 signature	F	Hydrosalpinx
	49	p53 signature	P	Hydrosalpinx
	71	p53 signature	D	Ovarian mucinous cystadenoma
	46	p53 signature	F	Uterine leiomyoma
	49	p53 signature	D	Uterine leiomyoma
	44	p53 signature	D	Uterine adenomatoid tumor
	48	p53 signature	F	Uterine endometrial endometrioid cancer
0	49	p53 signature	F	Uterine endometrial endometrioid cancer
1	68	p53 signatures (2)	D, F	Uterine endometrial endometrioid cancer
2	30	p53 signature	D	Uterine cervical squamous cell carcinoma
3	73	p53 signature	F	Uterine cervical squamous cell carcinoma
4	62	p53 signatures (2)	P, F	Ovarian clear cell carcinoma
5	68	p53 signature, STIL	D (p53 signature), F (STIL)	Ovarian high grade serous carcinoma
6	34	STIL	F	Hydrosalpinx
7	42	STIL	P	Hydrosalpinx
8	62	STIL	D	Tubal high grade serous carcinoma

### Ethical Issues

Ethical approval was obtained from Akita University, Faculty of Medicine, Ethics Committee (Reference No.1211). The data were analysed anonymously. The need of informed consent was waived by the committee because of the retrospective and noninvasive nature of the study.

### Immunohistochemical Analysis of Human Fallopian Tube

Sections of formalin-fixed paraffin-embedded fallopian tube tissue were cut into sections 4 μm thick and subjected to immunohistochemical staining via standard techniques using the Ventana Discovery XT® autostainer (Ventana Medical Systems Inc., Tucson, AZ, USA). Appropriate controls were included. Prior to this study, identification of p53 abnormalities had been performed by immunostaining of fallopian tube sections for p53 (1:100, anti-mouse, Clone DO-7; Novocastra Laboratories, Newcastle Upon Tyne, UK) and Ki67 (1:100, anti-mouse, Clone MIB-1; Dako, Glostrup, Denmark). For this study, additional deeper sections of tubal tissue containing p53 signatures were immunostained for an antibody reacting to vimentin (VMN) (1:100, anti-rabbit, Clone D21H3; Cell Signaling Technology, Inc., Danvers, MA, USA) and Filamin A (FLNA) (1:50, anti-rabbit, Clone Ab2; Sigma, St Louis, MO, USA).

### Immunofluorescence Analysis of Human Fallopian Tube

We performed immunofluorescence double staining for p53 and VMN to precisely evaluate VMN expression at the interface between p53 signature and adjacent normal tubal epithelium, and the interface between STIL and adjacent normal tubal epithelium. Antigen retrieval was performed using the Ventana Discovery® autostainer. We used primary antibodies against p53 (1:10, anti-mouse, Clone DO-7; Novocastra Laboratories) and VMN (1:50, anti-rabbit, Clone D21H3; Cell Signaling Technology). Goat anti-mouse Alexa-Fluor®-488- and goat anti-rabbit Alexa-Fluor®-546-conjugated antibodies (1:200; Life Technologies, Gaithersburg, MD, USA) were used as secondary antibodies. Finally, the slides were mounted with Vectashield and 4',6-diamidino-2-phenylindole mounting medium.

Confocal images that included the p53 signature/STIL and surrounding normal tubal epithelium were collected under a confocal microscope (LSM780; Zeiss, Göttingen, Germany) using a 63× objective lens. The image was imported into ZEN-LITE software (Zeiss), which allows manual delineation of the boundaries that define the compartments of interest and measurement of maximum intensity within the compartment.

### Immunohistochemical Analysis of mogp-TAg Mouse Fallopian Tube

We attempted to further investigate cell competition phenomenon between p53-transformed cells and normal cells in mammals using mogp-TAg mouse.The generation of mogp-TAg transgenic mouse is described in a previous literature.[18] We obtained formalin-fixed paraffin embedded sections of tubal tissue of mogp-TAg mouse, which is known to give rise to p53-altered lesions analogous to human STILs.[19,20] Immunohistochemistry for p53, Ki67, VMN and FLNA was performed via standard techniques using the Ventana Discovery XT® autostainer (Ventana Medical Systems Inc., Tucson, AZ, USA). The antibodies used were as follows: p53 (1:100, anti-rabbit, Clone FL-393; Santa Cruz Biotechnology, Inc., Texas, USA), Ki67 (1:400, anti-rabbit, Clone D3B5; Cell Signaling Technology, Inc., Danvers, MA, USA), VMIN (1:100, anti-rabbit, Clone D21H3; Cell Signaling Technology, Inc., Danvers, MA, USA) and FLNA (1:50, anti-rabbit, Clone Ab2; Sigma, St Louis, MO, USA). The study and use of animals were approved by Institutional Animal Care and Use Committee (IACUC).

### Statistical Analysis

Statistical analyses, including calculation of confidence interval, were performed using GraphPad PRISM 6.0 software (GraphPad Software, Inc., La Jolla, CA, USA).

## Results

### Immunohistochemical Analysis of Human Fallopian Tube

**Fig 3:**
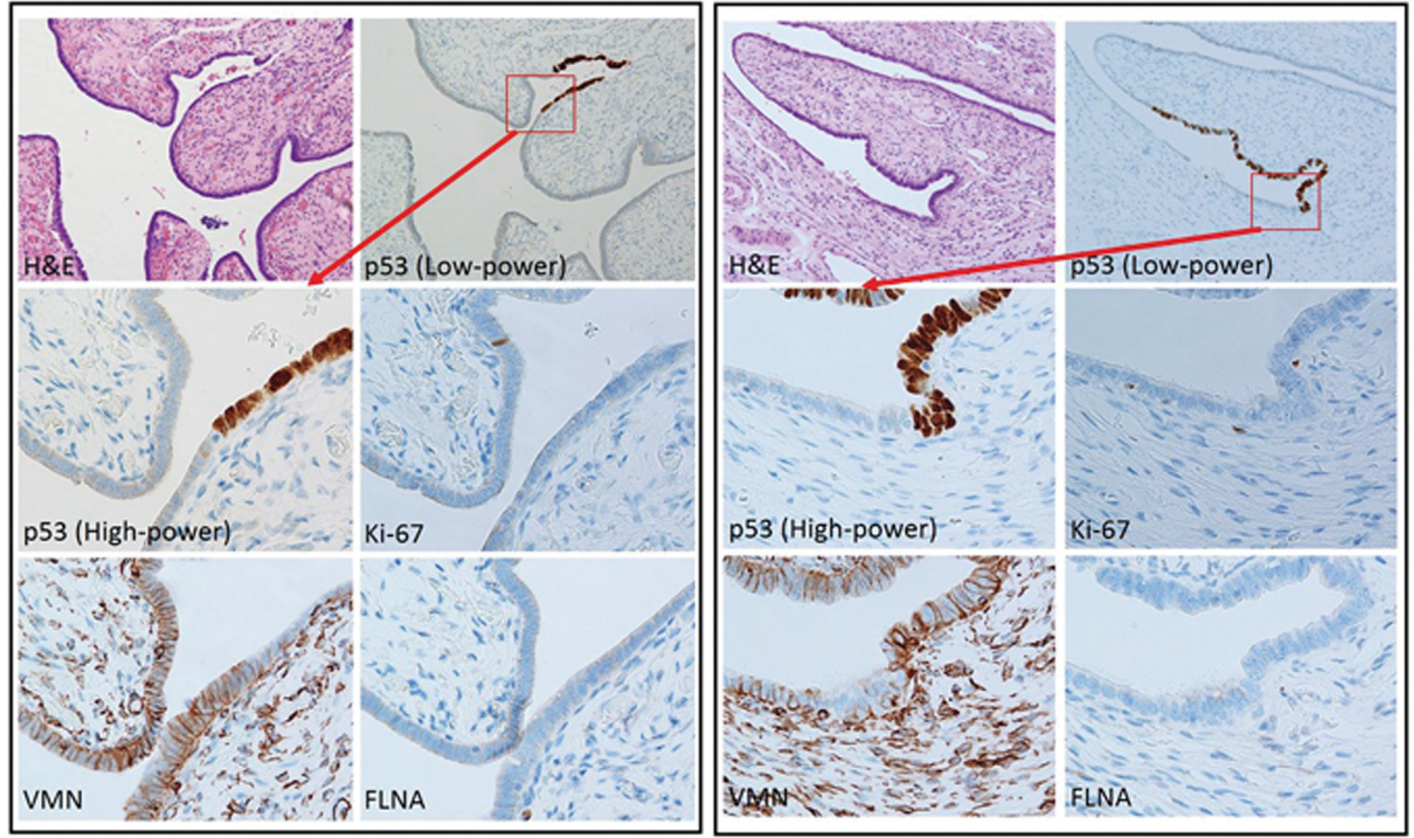
Histological and immunohistochemical features of fallopian tube epithelium containing p53 signatures. Two representative p53 signatures (right and left) are shown. The p53 signature is a small focus of linearly expanding p53-positive/p53 mutant cells that are morphologically bland. No increase in the Ki-67 labelling index was observed. VMN was expressed at variable intensity within the tubal epithelium, including p53 signatures. FLNA staining was negative or only faint.

**Fig 4:**
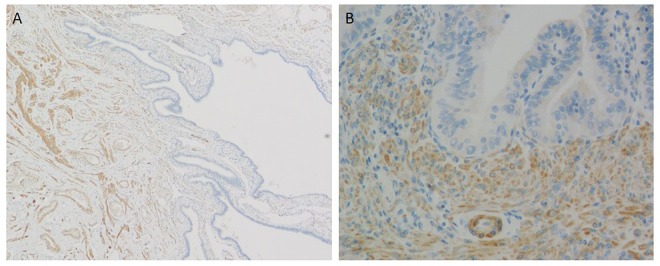
Filamin A expression in the human fallopian tube. A. low-power view, B. high-power view. Filamin A is expressed in smooth muscle bundles of the tubal wall. The immunoreactivity of the epithelium for filamin A is undetectable or only faint in the tubal epithelium.

Representative areas of tubal epithelium containing p53 signatures and their immunoreactivity are shown in Fig 3. The p53 signatures were located in an otherwise normal-appearing fallopian tube epithelium. By definition, neither cytological atypia nor an increase in Ki-67 labelling index was observed. VMN positivity was demonstrated at varying intensities throughout the tubal epithelium, including p53 signatures. Staining for FLNA was observed specifically in smooth muscle bundles of the tubal wall, and was undetectable or faint in the tubal epithelium. (Fig 4). Therefore, we focused specifically on VMN expression at the interface between p53-positive cells at the ends of the p53 signature/STIL where they are immediately adjacent to the p53-negative cells.

### Immunofluorescence Analysis of Human Fallopian Tube

Immunofluorescence double staining for p53 and VMN resulted in clear visualisation of 48 interfaces between p53-positive cells at the end of the p53 signature and adjacent p53-negative normal tubal epithelial cells in 28 fields, and 8 interfaces between p53-positive cells at the end of the STIL and adjacent p53-negative normal tubal epithelial cells in 6 fields. First, we measured labelling intensity for VMN at the interface between p53-positive cells at the end of the p53 signature/STIL and adjacent p53-negative normal tubal epithelial cells; this was designated as the VMN intensity at the interface (VI-I). The VMN intensities at the contact surfaces between background normal tubal epithelial cells were then measured and designated as VMN intensity of the background mucosa (VI-BG). The mean VI-BG in each field was calculated to define the standard VMN intensity (SVI). Finally, the VI-I/SVI and VI-BG/SVI ratios were generated for each intensity and expressed as the relative VMN index (RVI-I and RVI-BG, respectively).

**Fig 5:**
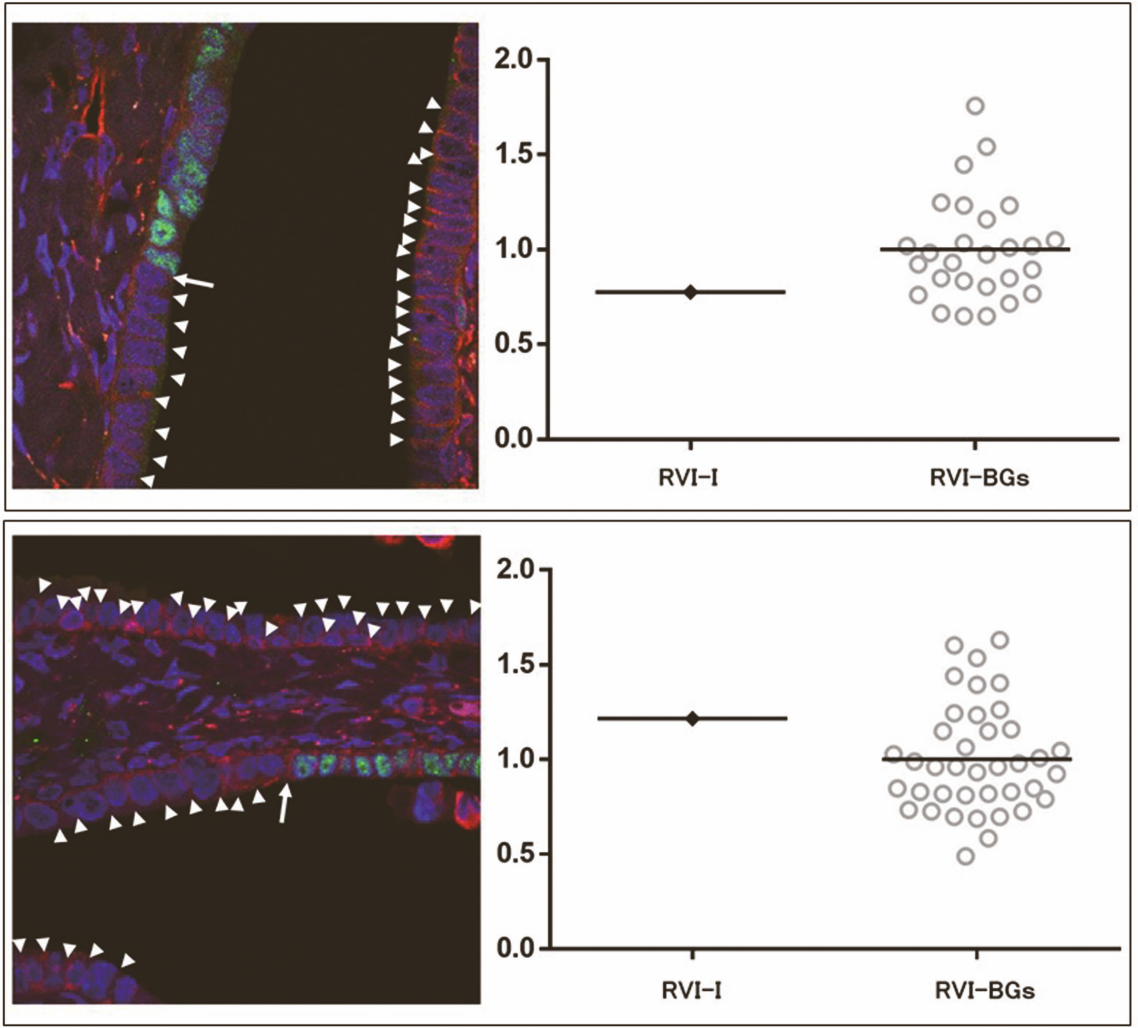
Immunofluorescent p53 (green)/VMN (red) double-staining images of the interface between p53 signature and adjacent normal tubal epithelium. VMN intensities at the interface between p53-positive cells and adjacent p53-negative normal tubal epithelial cells (arrow) and between background tubal epithelial cells (arrowhead) were measured. Graphs on the right show corresponding RVI-Is and RVI-BGs for each image.

**Fig 6:**
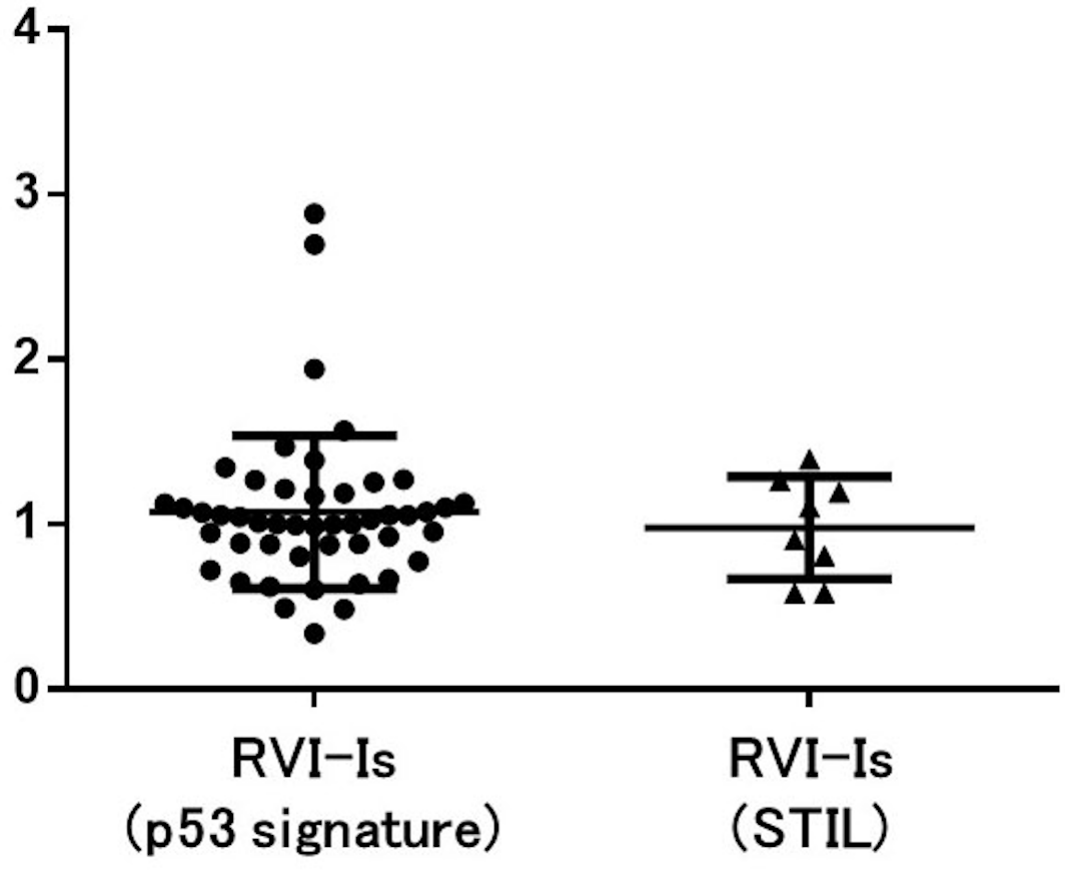
Relative VMN indices of the interfaces between p53 signatures/STILs and adjacent normal tubal epithelium (RVI-Is). The average RVI-I was 1.076 (95% CI, 0.9412 – 1.211) for p53 signature and 0.9790 (95% CI, 0.7206 – 1.237) for STIL.

The average number of VI-BGs obtained from each image was 26.0 for p53 signature and 20.2 for STIL. Representative immunofluorescence double staining images of the interface between p53 signature and adjacent normal tubal epithelium are shown in Fig 5, along with a graph showing the corresponding RVI-Is and RVI-BGs. The VI-BGs were variable. VI-I was almost always (> 93% of cases) below the maximum VI-BG. The average RVI-I was 1.076 (95% CI, 0.9412 – 1.211) for p53 signature and 0.9790 (95% CI, 0.7206 – 1.237) for STIL (Fig 6).

### Immunohistochemical Analysis of mogp-TAg Mouse Fallopian Tube

**Fig 7:**
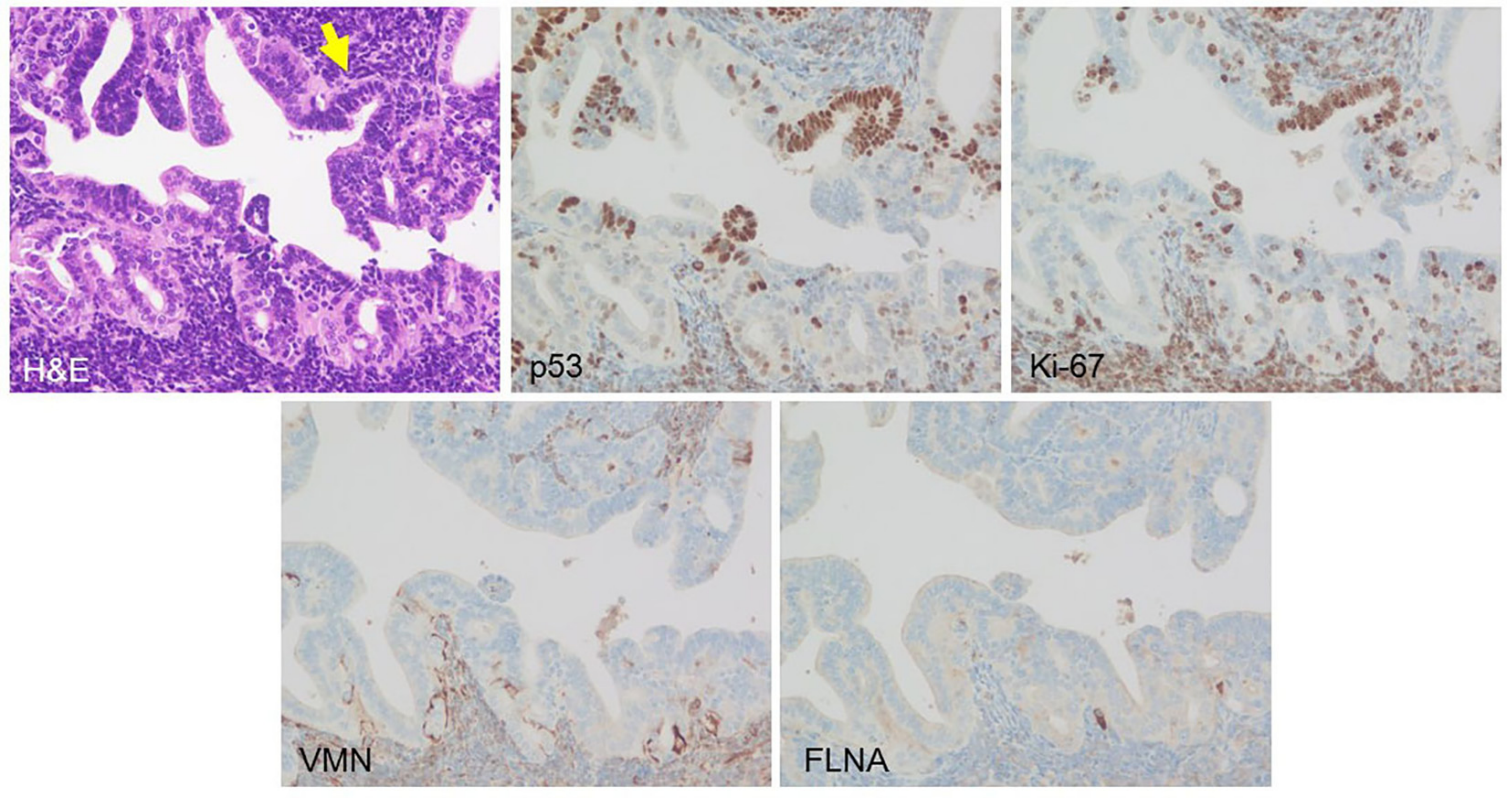
Linear expansion of p53-transformed cells in the fallopian tube of mogp-TAg mouse. In the tubal epithelium of mogp-TAg mouse, multiple linear expansions of p53-positive cells that showed no histological atypia or only subtle atypia were observed. These cells revealed high Ki-67 labelling index. VMN and FLNA expression in the tubal epithelium of mogp-TAg mouse was undetectable for the most part.

We found multiple foci of linearly expanding p53-positive cells that showed no histological atypia or only subtle atypia in the fallopian tube of mogp-TAg mouse (Fig 7). These cells revealed high Ki-67 labelling index. We considered these mouse tubal lesions rather analogous to human STILs. We found that VMN and FLNA expression in the tubal epithelium of mogp-TAg mouse were very focal and patchy. They were mostly negative in areas containing lesions with p53 alterations. Therefore, we did not move on to perform immunofluorescence analyses of the interfaces.

## Discussion

Over the past several decades, characterization of cancer cells themselves and investigation of the surrounding stroma/microenvironment have been the mainstream of cancer research. Recent advances in our understanding of cell competition phenomena and their roles in extrusion of mutant/transformed epithelial cells have brought attention to cell-to-cell interactions between mutant/transformed epithelial cells and adjacent normal epithelial cells.[3,4,8,21,22] This is a new and intriguing concept, as most cancer researchers, including pathologists, have overlooked the features and functions of adjacent normal epithelial cells.

Here, we assessed cell competition between normal epithelial cells and transformed epithelial cells in human tissue *in vivo*. This study was initiated based on the hypothesis that if cell competition is a ubiquitous and constant phenomenon in the human epithelium, upregulation of established cell competition markers, such as FLNA and VMN, should be observed at the interface between normal and various mutant cells, including those with mutations in *TP53*, *PIK3CA*, and *RB1*, etc., even within surgically resected human tissues. The main challenge in testing this hypothesis involved the *in vivo* localization of a small number of cells harboring cancer-associated mutations. Immunohistochemical detection of mutant cells is one way to achieve this goal. Although it is impossible to identify cells with RAS or Src mutations immunohistochemically, diffuse and strong nuclear immunostaining using anti-p53 antibodies can detect p53 mutant cell.[23] We focused on p53 signatures and STILs of the fallopian tube rather than serous tubal intraepithelial carcinoma or overt pelvic high-grade serous carcinoma (HGSC), because EDAC by cell competition is speculated to occur during the early phase of carcinogenesis. The p53 signature is a putative precursor of HGSC which has been reported to harbor TP53 mutations that are common in HGSC. ^14^ But, it has a much higher prevalence rate than HGSC.[15] This suggests that most p53 signatures do not evolve into HGSC, and some could be eliminated from the fallopian tube.

The results of immunofluorescence double staining for p53 and VMN clearly showed that constant VMN upregulation could not be demonstrated at the interface between p53 mutant tubal epithelial cells and adjacent normal cells. FLNA expression was not assessable for cell competition, because the tubal cells did not express FLNA. Briefly, we did not validate the cell competition phenomenon or the EDAC process in human epithelium containing cells with *TP53* alterations.

One possibility is that upregulation of VMN and FLNA is a specific, rather than ubiquitous, event that occurs in epithelial cells adjacent to RAS- or Src-transformed cells. There may be other EDAC-associated markers upregulated in cells adjacent to *TP53* mutant cells. It is also necessary to consider the limitation of this study associated with the use of surgically resected formalin-fixed paraffin-embedded tissue, because upregulation of VMN and FLNA could be a transient event that is not reproducible in histological sections. Finally, from our data, we cannot completely exclude the possibility that cell competition does not take place in humans.

However, we feel that cell competition can explain some of the processes involved in human epithelial defense against cancer. Further studies are necessary to identify a ubiquitous cell competition marker that can be applied to human histological specimens. Collaborations between clinicians and researchers from various fields, including pathology, molecular biology and genomics, are required to define cell competition in humans and to determine its clinical significance.

## References

[1] Morata G, Ripoll P. Minutes: mutants of drosophila autonomously affecting cell division rate. Dev Biol. 1975;42(2):211–21. 1116643 10.1016/0012-1606(75)90330-9

[2] Moreno E, Basler K. dMyc transforms cells into super-competitors. Cell. 2004;117(1):117–29. 15066287 10.1016/s0092-8674(04)00262-4

[3] Hogan C, Dupré-Crochet S, Norman M, Kajita M, Zimmermann C, Pelling AE, et al Characterization of the interface between normal and transformed epithelial cells. Nat Cell Biol. 2009;11(4):460–7. 10.1038/ncb1853 19287376

[4] Kajita M, Hogan C, Harris AR, Dupre-Crochet S, Itasaki N, Kawakami K, et al Interaction with surrounding normal epithelial cells influences signalling pathways and behaviour of Src-transformed cells. J Cell Sci. 2010;123(Pt 2):171–80. 10.1242/jcs.057976 20026643 PMC2954245

[5] Tamori Y, Bialucha CU, Tian AG, Kajita M, Huang YC, Norman M, et al Involvement of Lgl and Mahjong/VprBP in cell competition. PLoS Biol. 2010;8(7):e1000422 10.1371/journal.pbio.1000422 20644714 PMC2903597

[6] Norman M, Wisniewska KA, Lawrenson K, Garcia-Miranda P, Tada M, Kajita M, et al Loss of Scribble causes cell competition in mammalian cells. J Cell Sci. 2012;125(Pt 1):59–66. 10.1242/jcs.085803 22250205 PMC3269023

[7] Mamada H, Sato T, Ota M, Sasaki H. Cell competition in mouse NIH3T3 embryonic fibroblasts is controlled by the activity of Tead family proteins and Myc. J Cell Sci. 2015;128(4):790–803. 10.1242/jcs.163675 25588835

[8] Kajita M, Sugimura K, Ohoka A, Burden J, Suganuma H, Ikegawa M, et al Filamin acts as a key regulator in epithelial defence against transformed cells. Nat Commun. 2014;5:4428 10.1038/ncomms5428 25079702

[9] Chen CL, Schroeder MC, Kango-Singh M, Tao C, Halder G. Tumor suppression by cell competition through regulation of the Hippo pathway. Proc Natl Acad Sci U S A. 2012;109(2):484–9. 10.1073/pnas.1113882109 22190496 PMC3258595

[10] Tamori Y, Bialucha CU, Tian AG, Kajita M, Huang YC, Norman M, et al Involvement of Lgl and Mahjong/VprBP in cell competition. PLoS Biol. 2010;8(7):e1000422 10.1371/journal.pbio.1000422 20644714 PMC2903597

[11] Portela M, Casas-Tinto S, Rhiner C, López-Gay JM, Domínguez O, Soldini D, et al Drosophila SPARC is a self-protective signal expressed by loser cells during cell competition. Dev Cell. 2010;19(4):562–73. 10.1016/j.devcel.2010.09.004 20951347

[12] Petrova E, Soldini D, Moreno E. The expression of SPARC in human tumors is consistent with its role during cell competition. Commun Integr Biol. 2011;4(2):171–4. 10.4161/cib.4.2.14232 21655431 PMC3104570

[13] Levayer R, Hauert B, Moreno E. Cell mixing induced by myc is required for competitive tissue invasion and destruction. Nature. 2015;524(7566):476–80. 10.1038/nature14684 26287461

[14] Lee Y, Miron A, Drapkin R, Nucci MR, Medeiros F, Saleemuddin A, et al A candidate precursor to serous carcinoma that originates in the distal fallopian tube. J Pathol. 2007;211(1):26–35. 17117391 10.1002/path.2091

[15] Jarboe E, Folkins A, Nucci MR, Kindelberger D, Drapkin R, Miron A, et al Serous carcinogenesis in the fallopian tube: a descriptive classification. Int J Gynecol Pathol. 2008;27(1):1–9. 18156967 10.1097/pgp.0b013e31814b191f

[16] Vang R, Visvanathan K, Gross A, Maambo E, Gupta M, Kuhn E, et al Validation of an algorithm for the diagnosis of serous tubal intraepithelial carcinoma. Int J Gynecol Pathol. 2012;31(3):243–53. 10.1097/PGP.0b013e31823b8831 22498942 PMC3366037

[17] Visvanathan K, Vang R, Shaw P, Gross A, Soslow R, Parkash V, et al Diagnosis of serous tubal intraepithelial carcinoma based on morphologic and immunohistochemical features: a reproducibility study. Am J Surg Pathol. 2011;35(12):1766–75. 10.1097/PAS.0b013e31822f58bc 21989347 PMC4612640

[18] Miyoshi I, Takahashi K, Kon Y, Okamura T, Mototani Y, Araki Y, et al Mouse transgenic for murine oviduct-specific glycoprotein promoter-driven simian virus 40 large T-antigen: tumor formation and its hormonal regulation. Mol Reprod Dev. 2002;63(2):168–76. 12203826 10.1002/mrd.10175

[19] Sherman-Baust CA, Kuhn E, Valle BL, Shih IM, Kurman RJ, Wang TL, et al A genetically engineered ovarian cancer mouse model based on fallopian tube transformation mimics human high-grade serous carcinoma development. J Pathol. 2014;233(3):228–37. 10.1002/path.4353 24652535 PMC4149901

[20] Kobayashi Y, Kashima H, Wu RC, Jung JG, Kuan JC, Gu J, et al Mevalonate Pathway Antagonist Suppresses Formation of Serous Tubal Intraepithelial Carcinoma and Ovarian Carcinoma in Mouse Models. Clin Cancer Res. 2015;21(20):4652–62. 10.1158/1078-0432.CCR-14-3368 26109099 PMC4609247

[21] Wu SK, Gomez GA, Michael M, Verma S, Cox HL, Lefevre JG, et al Cortical F-actin stabilization generates apical-lateral patterns of junctional contractility that integrate cells into epithelia. Nat Cell Biol. 2014;16(2):167–78. 10.1038/ncb2900 24413434

[22] Ohoka A, Kajita M, Ikenouchi J, Yako Y, Kitamoto S, Kon S, et al EPLIN is a crucial regulator for extrusion of RasV12-transformed cells. J Cell Sci. 2015;128(4):781–9. 10.1242/jcs.163113 25609711

[23] Kuhn E, Kurman RJ, Vang R, Sehdev AS, Han G, Soslow R, et al TP53 mutations in serous tubal intraepithelial carcinoma and concurrent pelvic high-grade serous carcinoma—evidence supporting the clonal relationship of the two lesions. J Pathol. 2012;226(3):421–6. 10.1002/path.3023 21990067 PMC4782784

