# Correction: Expression of Cell Competition Markers at the Interface between p53 Signature and Normal Epithelium in the Human Fallopian Tube

**DOI:** 10.1371/journal.pone.0160136

**Published:** 2016-07-21

**Authors:** Masahiko Kito, Daichi Maeda, Yukitsugu Kudo-Asabe, Naoki Sato, Ie-Ming Shih, Tian-Li Wang, Masamitsu Tanaka, Yukihiro Terada, Akiteru Goto

In Table 1, the values in the Case column should be listed from 1 to 18. Please see the corrected Table 1 here.

**Table 1:** Clinicopathological features of cases evaluated in this study.

Case	Age	Lesions evaluated	Location(P; proximal fallopian tube, D; distal fallopian tube, F; fimbriated end)	Pelvic disease the surgery was performed for
1	32	p53 signature	D	Hydrosalpinx
2	41	p53 signature	F	Hydrosalpinx
3	44	p53 signature	F	Hydrosalpinx
4	49	p53 signature	P	Hydrosalpinx
5	71	p53 signature	D	Ovarian mucinous cystadenoma
6	46	p53 signature	F	Uterine leiomyoma
7	49	p53 signature	D	Uterine leiomyoma
8	44	p53 signature	D	Uterine adenomatoid tumor
9	48	p53 signature	F	Uterine endometrial endometrioid cancer
10	49	p53 signature	F	Uterine endometrial endometrioid cancer
11	68	p53 signatures (2)	D, F	Uterine endometrial endometrioid cancer
12	30	p53 signature	D	Uterine cervical squamous cell carcinoma
13	73	p53 signature	F	Uterine cervical squamous cell carcinoma
14	62	p53 signatures (2)	P, F	Ovarian clear cell carcinoma
15	68	p53 signature, STIL	D (p53 signature), F (STIL)	Ovarian high grade serous carcinoma
16	34	STIL	F	Hydrosalpinx
17	42	STIL	P	Hydrosalpinx
18	62	STIL	D	Tubal high grade serous carcinoma
